# LASSO-empowered nomogram integrating nutritional-inflammatory-tumor characteristics predicts immunotherapy outcomes in advanced HCC: Large retrospective cohort

**DOI:** 10.3389/fimmu.2025.1626940

**Published:** 2025-12-01

**Authors:** Shuifang Hu, Mingcong Xu, Shuping Li, Wei Chen, Zhenwei Peng

**Affiliations:** 1 Department of Radiation Oncology, The First Affiliated Hospital of Sun Yat-sen University, Guangzhou, China; 2 Center of Hepato-Pancreato-Biliary Surgery, The First Affiliated Hospital of Sun Yat-sen University, Guangzhou, China; 3 Department of Pancreaticobiliary Surgery, The First Affiliated Hospital of Sun Yat-sen University, Guangzhou, China; 4 Institute of Precision Medicine, The First Affiliated Hospital of Sun Yat-sen University, Guangzhou, China; 5 Cancer Center, The First Affiliated Hospital of Sun Yat-sen University, Guangzhou, China; 6 Clinical Trials Unit, The First Affiliated Hospital of Sun Yat-sen University, Guangzhou, China

**Keywords:** hepatocellular carcinoma, immunotherapy, nomogram, LASSO regression, prognostic biomarkers

## Abstract

**Background & Aims:**

Immune checkpoint inhibitors (ICIs) show heterogeneous efficacy in advanced hepatocellular carcinoma (HCC), but existing biomarkers are invasive and costly. We aimed to develop a noninvasive prognostic model using routine clinical parameters.

**Materials and methods:**

This retrospective study included 537 advanced HCC patients treated with PD-1/PD-L1 inhibitors, randomly divided into training (n=322) and validation (n=215) cohorts. Continuous variables were dichotomized using R packages. Univariate Cox regression followed by LASSO regression with 10-fold cross-validation selected predictive features for nomogram construction. Model performance was assessed via time-dependent receiver operating characteristic (ROC) curves, calibration plots, and decision curve analysis (DCA). Cox proportional hazards models identified independent prognostic factors.

**Results:**

Baseline characteristics were balanced between training and validation cohorts (P>0.05). The LASSO-derived nomogram incorporated 13 risk factors, which encompass multiple dimensions such as tumor characteristics, nutritional status, and inflammation. The model demonstrated robust discrimination, with the area under the curve (AUC) values exceeding 0.75 for 3-, 6-, 12-, and 24-month overall survival (OS). Calibration curves demonstrated a strong concordance between the predicted survival probabilities and the actual observations, and DCA revealed that the nomogram could increase net benefit. Additionally, the nomogram successfully stratified patients into low-risk and high-risk groups based on OS risk, with significant survival differences observed between the two groups in both the training and validation cohorts (all p < 0.001).

**Conclusions:**

This validated nomogram integrating inflammatory, nutritional, and tumor characteristics provides a cost-effective tool for prognostic stratification in advanced HCC patients undergoing immunotherapy, potentially guiding personalized therapeutic strategies.

## Introduction

According to the latest global cancer statistics, liver cancer is the third leading cause of cancer-related mortality, with approximately 757,000 deaths worldwide (1). Notably, China has a significantly higher incidence and mortality rate compared to the global average. Over 50% of patients present with advanced-stage disease at diagnosis due to nonspecific early symptoms, high Hepatitis B Virus (HBV) prevalence, and inadequate surveillance (2, 3).

However, durable responses to immune checkpoint blockade (ICB) in HCC remain restricted to a minority of patients, with existing biomarkers (e.g., PD-L1, TMB) demonstrating limited clinical utility for guiding precision therapy due to intratumoral heterogeneity, high detection costs, and inconsistent predictive performance (4–6). For instance, although PD-1/PD-L1 inhibitors have been approved by the FDA for TMB-high solid tumors, their response rates vary significantly across studies (15-30%), and their clinical application is constrained by inconsistent cutoff values (8–15 mutations/Mb) and lack of standardized detection methods (7). Therefore, there is an urgent need for non-invasive predictive tools based on routinely available clinical parameters to optimize treatment decisions.

Previous studies have shown that various biomarkers can affect the prognosis of immunotherapy in HCC patients, including markers related to tumor biology, nutritional status, and inflammatory metabolism. Among them, alpha-fetoprotein (AFP) is not only an important indicator for the diagnosis and prognosis of HCC, but its dynamic changes can also predict the efficacy of immunotherapy (8). The Prognostic Nutritional Index (PNI) has been confirmed as an independent predictor of OS in combination with immunotherapy (HR = 1.77, p < 0.001) (9, 10). Metabolic markers, such as lactate dehydrogenase (LDH), which reflects tumor glycolytic activity, are associated with poor prognosis when baseline levels are elevated (11). Fibrinogen (FIB) reduces the effectiveness of immunotherapy by promoting angiogenesis and creating an immunosuppressive microenvironment (12). Additionally, HCC is a malignancy closely related to chronic inflammation, and systemic inflammation affects the efficacy of ICIs by reshaping the tumor immune microenvironment (13). Inflammatory markers, such as the Systemic Immune-Inflammation Index (SII) and Neutrophil-Lymphocyte Ratio (NLR), are associated with poorer survival outcomes in HCC patients (14–18). However, there is no consensus on which biomarkers have the most predictive prognostic value, and the interactions between tumor characteristics, nutrition, and inflammatory metabolism still require systematic investigation.

To address fragmented biomarker studies and multicollinearity, we developed a multidimensional nomogram integrating pretreatment baseline data of nutritional, inflammatory, and tumor characteristics. Least Absolute Shrinkage and Selection Operator (LASSO) regression with cross-validation identified key predictors, enabling robust prognostic stratification. This model transcends single-biomarker limitations, offering a cost-effective, noninvasive tool to guide personalized immunotherapy in advanced HCC.

## Materials and methods

### Patient characteristics

**Figure 1:**
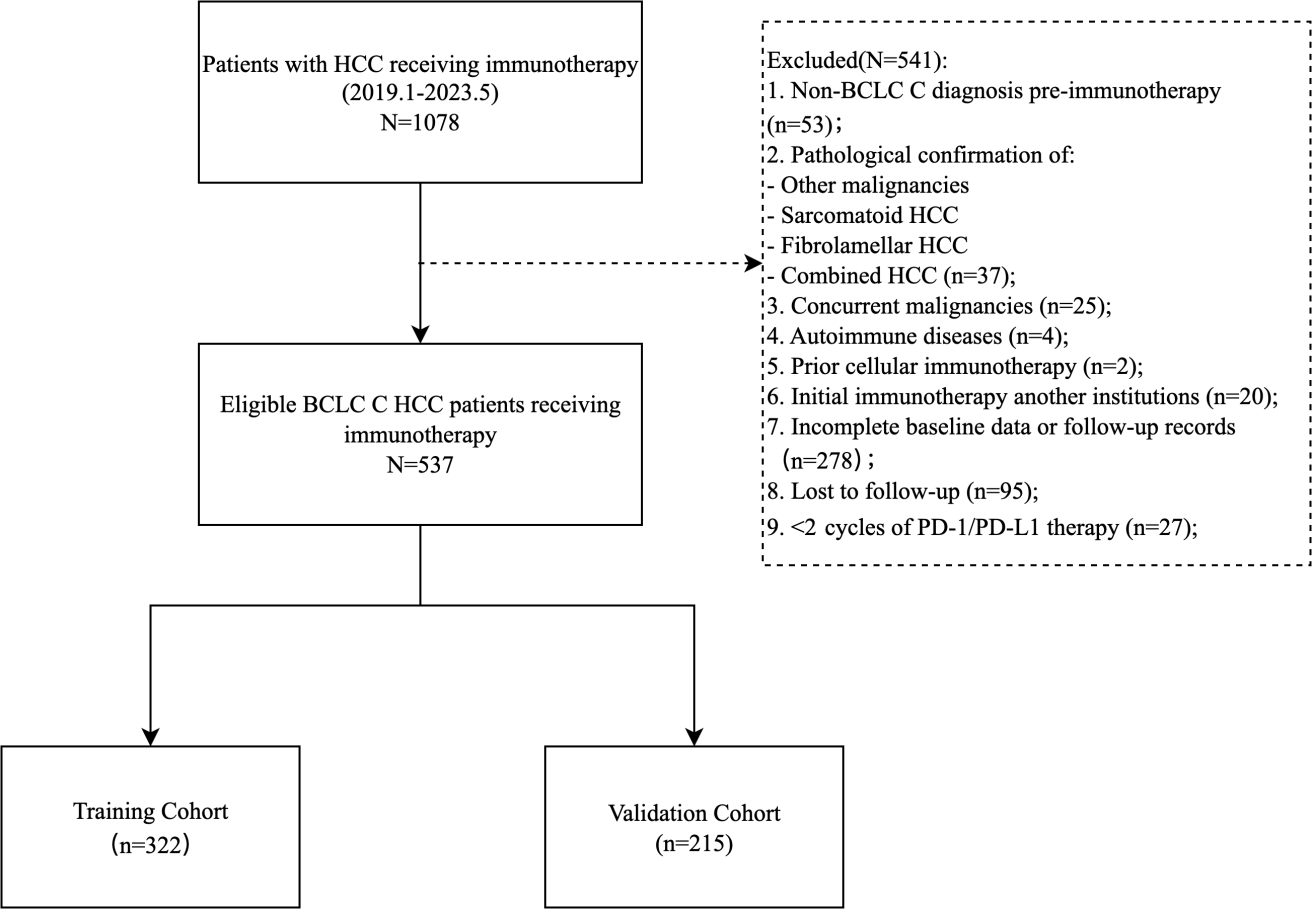
Flowchart of this study population.

This single-center retrospective cohort study enrolled 537 patients with Barcelona Clinic Liver Cancer (BCLC) C stage HCC who received PD-1/PD-L1 inhibitors (atezolizumab, camrelizumab, etc.) as monotherapy or combined with local therapies (TACE, HAIC, radiofrequency ablation), targeted agents (anti-angiogenics, TKIs), or radiotherapy at Sun Yat-sen University First Hospital (January 2019–May 2023). Inclusion criteria: (1) age ≥ 18 years; (2) histologically/radiologically confirmed unresectable or recurrent metastatic advanced HCC (BCLC C); (3) ≥ 2 cycles of PD-1/PD-L1 therapy; (4) ECOG performance score 0-1; (5) Child-Pugh A/B; (6) complete baseline clinical, imaging, and laboratory data. Exclusion criteria: (1) mixed/sarcomatoid HCC; (2) concurrent malignancies; (3) first immunotherapy administered outside our hospital; (4) missing key data or incomplete follow-up; (5) active autoimmune disease requiring systemic therapy. Patients were randomized 6:4 into training (n=322) and validation (n=215) cohorts (**Figure 1**).

This study was approved by the Institutional Research Ethics Committee of the First Affiliated Hospital of Sun Yat-sen University. Due to the retrospective nature, informed consent was waived. The study was conducted in accordance with the Helsinki Declaration.

### Data collection

Demographic characteristics, treatment-related details, and clinical baseline characteristics from the week prior to the first immunotherapy were collected through the electronic medical record system. Demographic data included age, gender, weight, height; etiology included smoking history, alcohol consumption history, hepatitis B virus (HBV) and hepatitis C virus (HCV) infections; imaging features included liver cirrhosis, lymph node metastasis, extrahepatic metastasis, portal vein tumor thrombus (PVTT), hepatic vein tumor thrombus (HVTT), and ascites; laboratory indicators included liver function [total bilirubin (TBIL), aspartate aminotransferase (AST), alanine aminotransferase (ALT), albumin (ALB), globulin (GLO)], blood routine [hemoglobin (Hb), platelet distribution width (PDW), neutrophil count, lymphocyte count, platelet count, monocyte count], AFP, LDH, and FIB. Based on these indicators, composite scores were calculated, including body mass index (BMI), NLR, platelet-to-lymphocyte ratio (PLR), lymphocyte-to-monocyte ratio (LMR), albumin-to-globulin ratio (AGR), PNI, SII, aminotransferase-to-platelet ratio index (APRI), albumin-bilirubin index (ALBI), aminotransferase-to-neutrophil ratio index (ANRI), Hb, albumin, lymphocyte, and platelet (HALP) score, and the modified Gustave Roussy Immune (GRIm). Standardized Formulas for Composite Scores were as follows:

BMI= Weight (kg)/Height (m) ^2^;
NLR= Neutrophil count/Lymphocyte count;
PLR= Platelet count/Lymphocyte count;
LMR= Lymphocyte count/Monocyte count;
AGR= ALB (g/L)/GLO (g/L);
PNI=Serum albumin (g/L)+5× lymphocyte count (×10^9^/L) (19);
SII= [Neutrophil count (×10^9^/L)×Platelet count (×10^9^/L)]/Lymphocyte count (×10^9^/L) (20);
APRI=[AST (U/L)/upper limit of normal range] × 100/Platelet count (×10^9^/L) (21);
ALRI=AST (U/L)/Lymphocyte count (×10^9^/L) (22);
ALBI=log_10_TBIL (umol/L)×0.66−ALB (g/L)×0.085 (23);
ANRI= AST (U/L)/Neutrophil count (×10^9^/L)​;
HALP= Hb (g/L)×ALB (g/L)×Lymphocyte count (×10^9^/L)/Platelet count (×10^9^/L) (24);
GRIm-score:NLR>6, ALB<35g/L, LDH>240U/L, 1 point for each, total score range: 0-3.

### Follow-up

Patients were assessed every 2–3 months during the first year, and every 6 months thereafter. Follow-up evaluations included routine blood tests, liver function tests, AFP levels, and CT/MRI scans. Two radiologists independently analyzed the imaging results, and in case of any discrepancies, the final decision was made through discussion or by consulting a third-party expert. OS was defined as the time from the start of immunotherapy to death from any cause or the last follow-up, while progression-free survival (PFS) was defined as the time from the start of immunotherapy to radiological progression (RECIST 1.1), death, or the last follow-up. The follow-up period ended on May 30, 2023.

### Statistical analysis

Normality of continuous variables was assessed using the Shapiro-Wilk test. Normally distributed variables were expressed as mean ± standard deviation and compared via Student’s t-test, while non-normally distributed variables were reported as median (interquartile range [IQR]) and analyzed using the Mann-Whitney U test. Categorical variables were presented as frequencies (percentages) and compared via Pearson’s χ² test or Fisher’s exact test, as appropriate. Optimal cutoff values for continuous biomarkers were determined using R packages based on OS. Univariate Cox proportional hazards regression was first performed to identify potential prognostic factors (P < 0.05 threshold for inclusion). Variables meeting this criterion were subsequently incorporated into multivariate Cox regression. To address multicollinearity and enhance model robustness, LASSO regression with 10-fold cross-validation was used to optimize variable selection. The optimal penalty parameter (λ) was determined using the λ.1se criterion, which selects the most parsimonious model whose performance is within one standard error of the minimum cross-validated error. This approach prioritizes model simplicity and generalizability over maximal fitting of the training data. Multicollinearity among the LASSO-selected variables was explicitly assessed by calculating variance inflation factors (VIFs). All VIF values remained below the conservative threshold of 5, confirming that significant multicollinearity was not present in the final model. A prognostic nomogram was then constructed using selected predictors. Discrimination was evaluated using time-dependent ROC curves. Calibration curves assessed agreement between predicted and observed survival probabilities. Clinical utility was quantified via DCA. Risk stratification was performed using the median value of calculated risk scores in the training cohort as the cutoff. This threshold was then applied to the validation cohort to categorize patients into high- and low-risk groups. Survival differences across risk strata were visualized using Kaplan-Meier curves and compared via log-rank tests. All analyses were conducted using SPSS 26.0 and R 4.3.1 (packages: survival, ggplot2, time-ROC.etc). A p-value < 0.05 was considered statistically significant.

## Results

### Patient characteristics

The study ultimately included 537 patients, with 322 in the training group and 215 in the validation group (**Figure 1**). The median PFS was 7.7 months (95% CI: 6.5-9.7), and the median OS was 27.3 months (95% CI: 21.9-35.0) (**Supplementary Figure S1**).

Baseline demographic and clinical characteristics are summarized in **Table 1**. The cohort comprised predominantly males (91.1%), with a median age of 53.31 ± 11.17 years and median BMI of 22.87 ± 3.31 kg/m². The majority (85.1%) had HBV infection, while HCV infection was rare (3.4%). Imaging studies demonstrated cirrhosis in 40.6% of cases, PVTT in 50.1%, and extrahepatic metastases in 54.0%. Hepatic function assessments classified 77.7% as Child-Pugh grade A and 76.2% as ALBI grade 2. Elevated AFP levels exceeding 400 ug/L were observed in 50.8% of patients. The most commonly used immunotherapy agents are camrelizumab (36.1%) and tislelizumab (29.4%). The training and validation cohorts demonstrated balanced baseline characteristics across all key variables, with no statistically significant intergroup differences (all p > 0.05).

**Table 1:** Baseline characteristics of enrolled HCC patients in training set and validation set.

Characteristics	Total (n=537)	Training Group (n=322)	Validation Group (n=215)	*P-*value
Gender				0.311
Male	489 (91.1%)	297 (92.2%)	192 (89.3%)	
Female	48 (8.9%)	25 (7.8%)	23 (10.7%)	
Age (years)	53.31 ± 11.17	53.50 ± 11.35	53.03 ± 10.92	0.629
BMI (kg/m²)	22.87 ± 3.31	22.93 ± 3.28	22.77 ± 3.35	0.592
Smoking				1
No	308 (57.4%)	185 (57.5%)	123 (57.2%)	
Yes	229 (42.6%)	137 (42.5%)	92 (42.8%)	
Alcohol Consumption				0.774
No	392 (73.0%)	237 (73.6%)	155 (72.1%)	
Yes	145 (27.0%)	85 (26.4%)	60 (27.9%)	
HVTT				0.973
None	473 (88.1%)	283 (87.9%)	190 (88.4%)	
Present	64 (11.9%)	39 (12.1%)	25 (11.6%)	
PVTT				1
None	268 (49.9%)	161 (50.0%)	107 (49.8%)	
Present	269 (50.1%)	161 (50.0%)	108 (50.2%)	
Lymph Node Metastasis				0.421
None	369 (68.7%)	226 (70.2%)	143 (66.5%)	
Present	168 (31.3%)	96 (29.8%)	72 (33.5%)	
Extrahepatic Metastasis				0.438
None	247 (46.0%)	153 (47.5%)	94 (43.7%)	
Present	290 (54.0%)	169 (52.5%)	121 (56.3%)	
HbsAg positive				0.705
No	80 (14.9%)	50 (15.5%)	30 (14.0%)	
Yes	457 (85.1%)	272 (84.5%)	185 (86.0%)	
HCV-Ab positive				0.404
No	519 (96.6%)	309 (96.0%)	210 (97.7%)	
Yes	18 (3.4%)	13 (4.0%)	5 (2.3%)	
Liver Cirrhosis				0.827
None	319 (59.4%)	193 (59.9%)	126 (58.6%)	
Present	218 (40.6%)	129 (40.1%)	89 (41.4%)	
Child-Pugh Grade				0.249
Class A	417 (77.7%)	256 (79.5%)	161 (74.9%)	
Class B	120 (22.3%)	66 (20.5%)	54 (25.1%)	
ALBI Grade				0.748
Grade 1	107 (19.9%)	62 (19.3%)	45 (20.9%)	
Grade 2	409 (76.2%)	246 (76.4%)	163 (75.8%)	
Grade 3	21 (3.9%)	14 (4.3%)	7 (3.3%)	
AFP (ng/mL)				0.503
≤ 400	264 (49.2%)	154 (47.8%)	110 (51.2%)	
> 400	273 (50.8%)	168 (52.2%)	105 (48.8%)	
Type of Immunotherapy				0.166
Atezolizumab	1 (0.2%)	0 (0.0%)	1 (0.5%)	
Adebelimumab	44 (8.2%)	26 (8.1%)	18 (8.4%)	
Camrelizumab	194 (36.1%)	115 (35.7%)	79 (36.7%)	
Durvalumab	1 (0.2%)	1 (0.3%)	0 (0.0%)	
Nivolumab	3 (0.6%)	1 (0.3%)	2 (0.9%)	
Pembrolizumab	27 (5.0%)	19 (5.9%)	8 (3.7%)	
Penpulimab	3 (0.6%)	1 (0.3%)	2 (0.9%)	
Sintilimab	94 (17.5%)	47 (14.6%)	47 (21.9%)	
Tislelizumab	158 (29.4%)	106 (32.9%)	52 (24.2%)	
Toripalimab	12 (2.2%)	6 (1.9%)	6 (2.8%)	

AFP, Alpha-fetoprotein; ALBI, Albumin-Bilirubin; BMI, Body Mass Index; HCV, Hepatitis C virus; HCC, Hepatocellular Carcinoma; HVTT, Hepatic Venous Tumor Thrombus; PVTT, Portal Venous Tumor Thrombus.

### Determination of optimal cut-off

The optimal cutoff values for baseline prognostic biomarkers associated with OS were determined based on the highest log-rank statistic (**Supplementary Figure S2**), including the following: age (38 years), BMI (19.78 kg/m²), AGR (0.94), LDH (191.00 U/L), FIB (3.13 g/L), Hb (128.00 g/L), PDW (10.50 fl), NLR (3.34), SII (1356.42), PLR (228.96), LMR (3.77), PNI (43), ANRI (27.01), ALRI (42.36), APRI (1.26), and HALP (17.52). Patients were classified into high-risk and low-risk groups based on these cutoff values for OS (**Supplementary Figure S3**).

### Univariate and multivariate Cox regression analysis

Through univariate Cox regression analysis in the training cohort, we identified 21 clinical factors significantly associated with OS (P < 0.05) (**Table 2**).

**Table 2:** Univariate and multivariate cox hazards analysis for overall survival in the training cohort.

Parameter	OS					
	Univariate			Multivariate		
	HR	95% CI	P-value	HR	95% CI	P-value
Gender, male vs female	0.73	0.34-1.57	0.4171			
Age, ≤38 vs >38	2.09	1.22-3.57	**0.0069**	1.92	1.04-3.56	**0.0384**
BMI, ≤19.78 vs >19.78	1.82	1.11-2.98	**0.0171**	1.66	0.96-2.88	0.0716
Smoking, yes vs no	0.91	0.61-1.36	0.6566			
Drinking, yes vs no	0.82	0.51-1.32	0.4218			
HVTT, yes vs no	1.70	1.01-2.86	**0.0476**	1.26	0.72-2.22	0.4208
PVTT, yes vs no	1.60	1.08-2.37	**0.0193**	1.05	0.65-1.69	0.8424
Lymphatic metastasis, yes vs no	1.04	0.68-1.59	0.8691			
Extrahepatic metastasis, yes vs no	0.99	0.67-1.47	0.9570			
HBV, positive vs negative	0.94	0.57-1.54	0.8012			
HCV, positive vs negative	1.33	0.54-3.27	0.5403			
Liver cirrhosis, yes vs no	1.85	1.25-2.75	**0.0022**	1.72	1.09-2.70	**0.0195**
Child-Pugh, B vs A	2.43	1.59-3.72	**<0.001**	1.12	0.63-1.99	0.7048
AFP, ≤400ng/ml vs > 400ng/ml	0.62	0.42-0.93	**0.0203**	0.69	0.44-1.10	0.1198
AGR, ≤0.94 vs >0.94	1.82	1.18-2.81	**0.0064**	1.01	0.62-1.67	0.9601
LDH, ≤191vs >191	0.43	0.25-0.73	**0.0018**	0.51	0.28-0.93	**0.0276**
FIB, ≤3.13 vs >3.13	0.64	0.43-0.95	**0.0287**	0.74	0.46-1.17	0.1917
Hb, ≤128 vs >128	1.78	1.2-2.62	**0.0038**	1.71	1.11-2.63	**0.0151**
PDW, ≤10.5 vs >10.5	0.57	0.29-1.09	0.0891			
NLR, ≤3.34 vs >3.34	0.64	0.43-0.95	**0.0273**	1.22	0.68-2.17	0.5076
SII, ≤1356.42 vs >1356.42	0.51	0.31-0.84	**0.0075**	0.66	0.30-1.47	0.3097
PLR, ≤228.96 vs >228.96	0.52	0.33-0.83	**0.0065**	0.72	0.34-1.53	0.3883
LMR, ≤3.77 vs >3.77	1.83	1.02-3.28	**0.0424**	1.15	0.59-2.28	0.6796
PNI, ≤43 vs >43	2.25	1.48-3.41	**<0.001**	1.26	0.73-2.18	0.4003
ANRI, ≤27.01 vs >27.01	0.60	0.40-0.91	**0.0157**	1.77	0.97-3.25	0.0646
ALRI,≤42.36 vs >42.36	0.50	0.33-0.74	**<0.001**	1.32	0.67-2.61	0.4248
APRI, ≤1.26 vs >1.26	0.47	0.32-0.70	**<0.001**	0.44	0.23-0.85	**0.0149**
HALP, ≤17.52 vs >17.52	2.40	1.53-3.76	**<0.001**	1.32	0.62-2.83	0.4698
ALBI, class 2/3 vs class 1	1.70	0.98-2.95	0.0602			
GRIm-Score, 2/3 vs 0/1	1.79	1.19-2.69	**0.0051**	0.91	0.53-1.58	0.7479
TACE, No vs Yes	0.95	0.63-1.43	0.8100			
Ablation, No vs Yes	1.16	(0.51-2.65)	0.7257			
Radiotherapy, No vs Yes	1.11	(0.58-2.13)	0.7589			

The bold text means P<0.05.

AFP, Alpha-fetoprotein; AGR, Albumin-to-Globulin Ratio; ALBI, Albumin-bilirubin Index; ALRI, Aminotransferase-to -lymphocyte Ratio Index; ANRI, Aminotransferase-to-neutrophil Ratio Index; APRI, AST-to-Platelet Ratio Index; BMI, Body Mass Index; CI, Confidence Interval; FIB, Fibrinogen; GRIm-Score, Modified Gustave Roussy Immune Score; HALP, Hemoglobin, Albumin, Lymphocyte, and Platelets; Hb, Hemoglobin; HBV, hepatitis B virus; HCV, hepatitis C virus; HR, Hazard Ratio; HVTT, Hepatic Venous Tumor Thrombus; LDH, Lactate Dehydrogenase; LMR, Lymphocyte-to-Monocyte Ratio; NLR, Neutrophil-Lymphocyte Ratio; PDW, Platelet Distribution Width; OS, Overall Survival; PLR, Platelet-to-Lymphocyte Ratio; SII, Systemic Immune-Inflammation Index; PNI, Prognostic Nutritional Index; PVTT, Portal Venous Tumor Thrombus; SII, Systemic Immune-Inflammation Index.

In the multivariate Cox regression model, LDH ≤ 191 U/L (HR = 0.51, 95% CI: 0.28-0.93, P = 0.028) and APRI ≤1.26 (HR = 0.44, 95% CI: 0.23-0.85, P = 0.015) demonstrated significant survival-protective effects, while Liver cirrhosis (HR = 1.72, 95% CI: 1.09-2.70, P = 0.020), Age ≤38 years (HR = 1.92, 95% CI: 1.04-3.56, P = 0.038) and Hb ≤ 128 g/L (HR = 1.71, 95% CI: 1.11-2.63, P = 0.015) was independently associated with a shortened OS (**Table 2**). Kaplan-Meier survival analysis for OS further validated the prognostic value of these biomarkers (**Supplementary Figure S4**).

### Prognostic factor selection and nomogram construction

**Figure 2:**
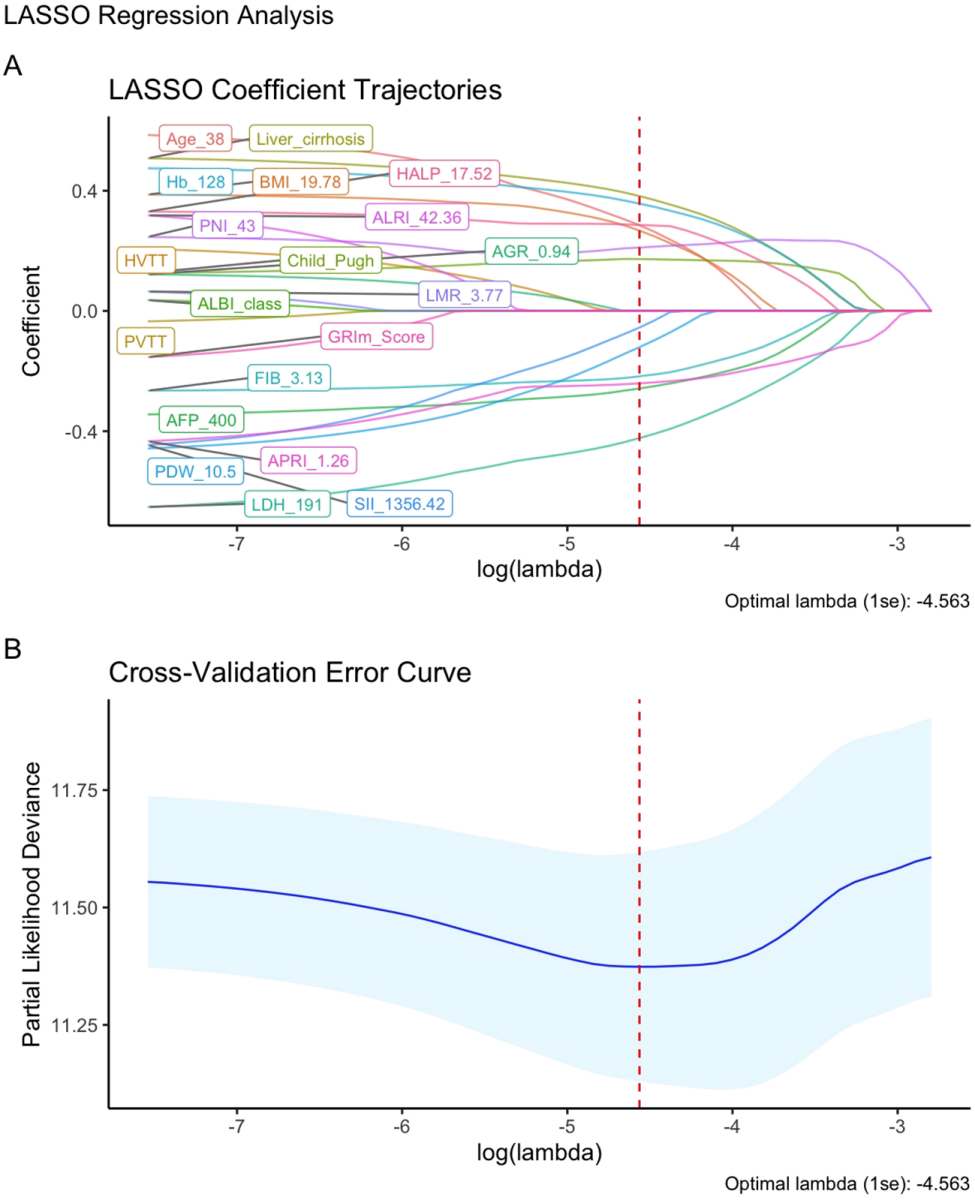
Feature Selection using LASSO regression. **(A)** LASSO coefficient path for OS-related potential prognostic factors; **(B)** LASSO regression cross-validation curve.

**Figure 3:**
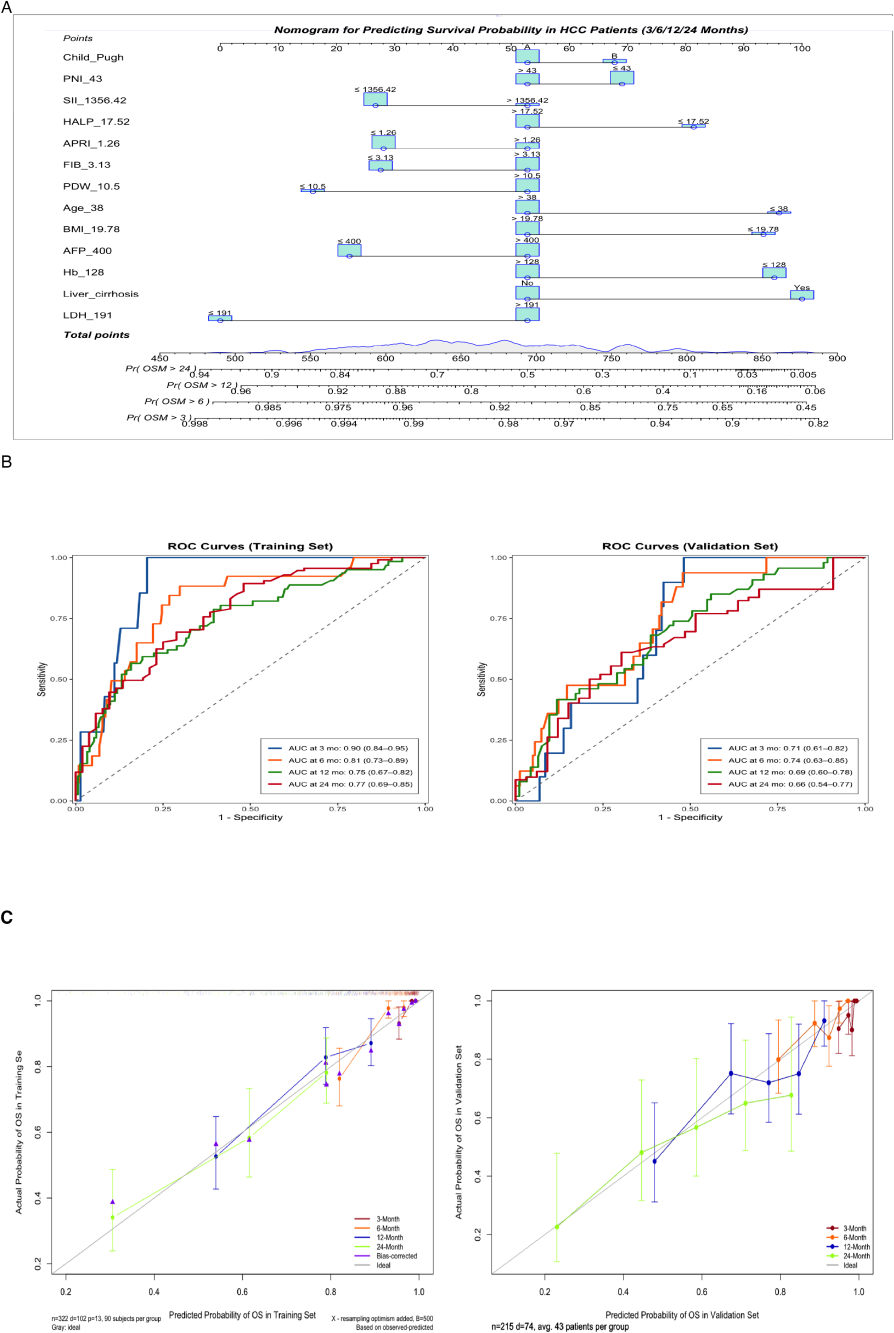
**(A)** Nomogram for predicting 3-, 6-, 12-, and 24-month overall survival. Instructions: Locate the patient’s value for each variable, draw a line upward to determine the points, sum all points, and locate the total points on the ‘Total Points’ axis. A line drawn downward to the survival axes indicates the predicted probability of survival at each timepoint. **(B)** Time-dependent receiver operating characteristic (ROC) curves at 3, 6, 12, and 24 months for predicting overall survival probabilities in the training and validation cohorts. **(C)** Calibration curves for predicting 3-, 6-, 12-, and 24-month survival probabilities in the training and validation sets.

Univariate Cox regression analysis (with a screening threshold of P < 0.2) was first performed to identify prognostic factors associated with OS. Subsequently, LASSO regression with 10-fold cross-validation and the λ.1SE criterion was used to optimize the variable selection. Thirteen independent prognostic factors with non-zero coefficients were identified: age, BMI, Child-Pugh grade, liver cirrhosis, AFP, FIB, Hb, LDH, PDW, PNI, SII, HALP and APRI (**Figure 2**; **Supplementary Figure S5**, **Supplementary Table S1**). The nomogram constructed based on these variables allows for the intuitive quantification of the contribution of each clinical factor to predicting the 3/6/12/24-month survival probability of advanced HCC patients after immunotherapy (**Figure 3A**).

### Model performance validation

**Figure 4:**
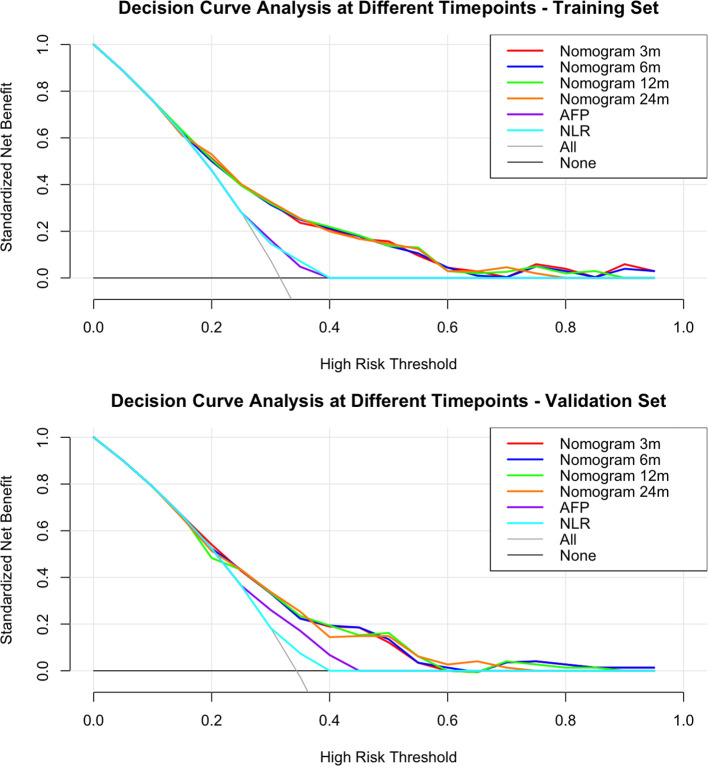
Decision curve analysis (DCA) of overall survival in the training and validation cohorts. The DCA compares the net clinical benefit of the nomogram, “Treat All,” “Treat None,” and traditional biomarkers.

The nomogram demonstrated good discriminatory ability in the training cohort, with a C-index of 0.72 (95% CI: 0.67–0.78). Time-dependent ROC curve analysis showed that the model predicted the 3/6/12/24-month OS with AUC values of 0.90 (95% CI: 0.84–0.95), 0.81 (95% CI: 0.73–0.89), 0.75 (95% CI: 0.67–0.82), and 0.77 (95% CI: 0.69–0.85) in the training cohort, respectively. The corresponding AUC values in the validation cohort remained stable (0.71 [95% CI:0.61–0.82], 0.74 [95% CI:0.63–0.85], 0.69 [95% CI:0.60–0.78], 0.66 [95% CI:0.54–0.77]) (**Figure 3B**). Calibration curves demonstrated a high concordance between the predicted survival probabilities and the Kaplan-Meier observed values in both the training and validation cohorts (**Figure 3C**). DCA further confirmed its clinical utility: when the threshold probability ranged from 20% to 60%, the nomogram showed a significantly higher net clinical benefit compared to traditional single biomarker models in both the training and validation cohorts (**Figure 4**).

### Survival analysis based on risk stratification from the nomogram

**Figure 5:**
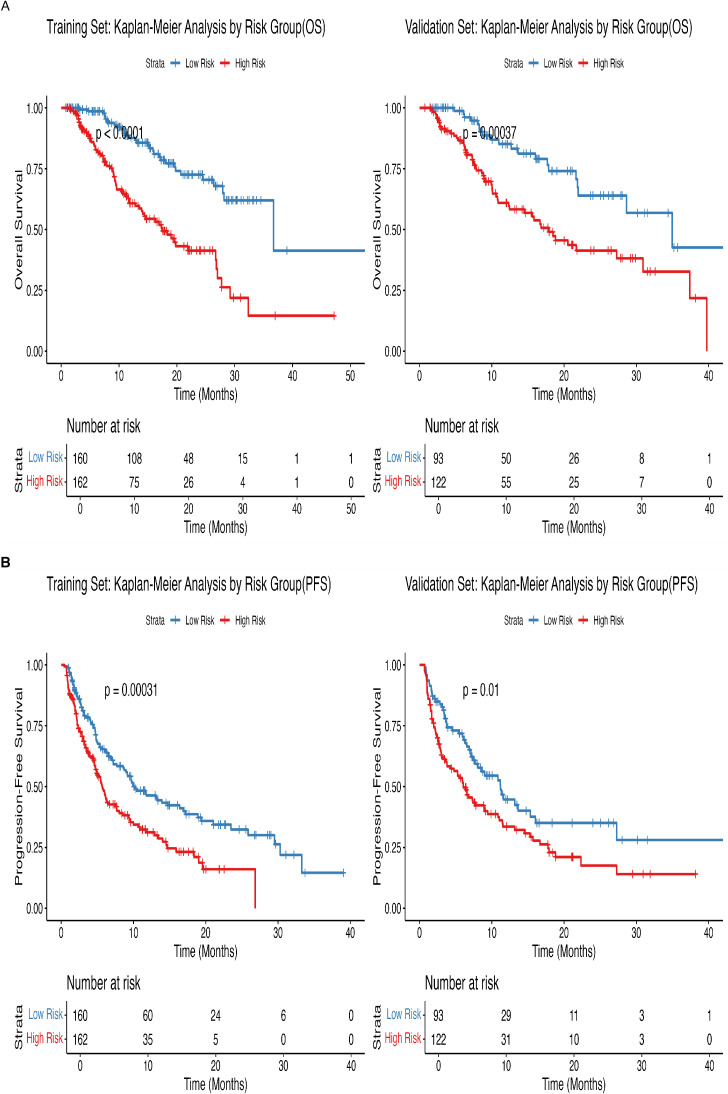
Kaplan-Meier plots of OS for the low-risk group and high-risk group in the training and validation cohort.

Using the nomogram developed in this study, patients were categorized into low-risk and high-risk groups based on calculated risk factors. In the training cohort, the high-risk group had an OS hazard ratio (HR) of 3.22 (95% CI: 2.11–4.91; P < 0.0001) (**Figure 5**). In the validation cohort, the HR for OS in the high-risk group was 2.47 (95% CI: 1.48–4.13; P < 0.001). While the nomogram was developed for OS, it also predicts PFS with clinical relevance. In the training cohort, the HR for PFS in the high-risk group was 1.69 (95% CI: 1.27–2.26; P < 0.001) (**Figure 5**), and in the validation cohort, the HR for PFS was 1.59 (95% CI: 1.11–2.28; P = 0.01).

### Subgroup validation demonstrating model robustness

The model exhibited consistent discriminative ability across different treatment strategies. For the ICI + anti-angiogenic therapy group (n=75), the AUCs at 3, 6, 12, and 24 months were 0.78 (95% CI: 0.67–0.88), 0.95 (0.86–1.00), 0.65 (0.45–0.86), and 0.66 (0.40–0.93), respectively. In the ICI + TKIs group (n=342), the corresponding AUCs were 0.85 (0.77–0.92), 0.78 (0.70–0.86), 0.73 (0.65–0.80), and 0.76 (0.67–0.85) (**Supplementary Figure S6**). There were no significant differences in OS (P = 0.91) or PFS (P = 0.76) between the treatment groups, confirming that the model’s risk stratification remained consistent regardless of the therapeutic regimen (**Supplementary Figure S7**).

In the HBV-positive cohort (n=457, 85.1%), the model maintained stable performance, with AUCs of 0.78 (0.70–0.86), 0.79 (0.72–0.86), 0.75 (0.69–0.81), and 0.74 (0.66–0.81) at 3, 6, 12, and 24 months. Calibration curves showed excellent alignment with observed outcomes, and decision curve analysis demonstrated significant net benefit within clinical decision thresholds, further validating the model’s clinical utility in HBV-endemic populations (**Supplementary Figure S8**).

## Discussion

Breakthrough advances in immunotherapy have significantly improved survival in advanced HCC patients, but the heterogeneity of treatment responses remains a challenge in clinical practice (25). There is a need for prognostic tools that integrate multiple biological factors to overcome the limitations of single biomarkers in predicting therapeutic efficacy. This study developed the first prognostic nomogram, constructed using 13 key indicators identified by LASSO regression, which integrates demographic, tumor, inflammatory, and nutritional metabolism features, providing a novel strategy for stratified management of advanced HCC in immunotherapy.

The 13 factors were categorized into four groups: (1) Demographic characteristics: Age, BMI; (2) Tumor characteristics and liver function: liver cirrhosis, AFP, Child Pugh; (3) Systemic inflammatory and fibrotic response: LDH, FIB, SII, HALP, APRI, PDW. These markers indicate tumor-associated inflammation and liver fibrosis progression (26–29); (4) Nutritional metabolism: Hb, PNI. The model demonstrated good discrimination and calibration in both the training and validation cohorts, with notable advantages in early prediction. Notably, the model demonstrated consistent predictive performance in both HBV-positive patients and across subgroups receiving different combination therapies, further validating its clinical applicability.

Among all factors, pretreatment elevated LDH conferred the highest weight in our model, underscoring its pivotal role. Our findings align with a large propensity score-matched study where high LDH levels (>241 U/L) were independently associated with shorter OS (median OS: 10.7 *vs*. 38.6 months, HR = 1.37, 95% CI: 1.20–1.55, P < 0.001) (11). Another retrospective study on patients undergoing HCC liver resection further confirmed that LDH is an independent risk factor for OS (HR = 1.807, 95% CI: 1.262–2.587, P < 0.001) (30). The prognostic value of LDH is closely related to its role in lactate metabolism. As a key rate-limiting enzyme in glycolysis, LDH catalyzes the conversion of pyruvate to lactate, driving tumor microenvironment acidification. This induces M2 macrophage polarization, inhibits CTL/NK cell function, and while also enhances Treg immunosuppressive activity, collectively shaping an immunosuppressive “cold tumor” microenvironment (31). Therefore, targeting the LDH-lactate axis may be a promising strategy to improve HCC immunotherapy response by reversing microenvironment acidification and restoring immune cell function.

As the end-stage manifestation of liver fibrosis (32), cirrhosis demonstrated significant prognostic value in our study. LASSO regression analysis revealed a significantly increased risk score in cirrhotic patients (coefficient +0.382, second only to LDH), indicating cirrhosis as an independent risk factor affecting immunotherapy efficacy. This finding, along with other predictive factors in our model, such as Child-Pugh (which incorporates cirrhosis assessment), FIB, and APRI, collectively forms a liver disease-based predictive framework for immunotherapy response. The poor outcomes may result from cirrhosis-induced immunosuppression (33), including dysfunctional macrophage/monocyte accumulation, impaired NK cell activity (34), and TGF-β-mediated expansion of MDSCs and Tregs (35). These findings underscore the importance of considering liver fibrosis status when selecting patients for immunotherapy.

As a classic biomarker for HCC, AFP is also an important independent predictor in the prognostic model of this study. This finding aligns with a study investigating the efficacy of immunotherapy in unresectable HCC, which identified a ≥20% reduction in AFP within 8 weeks as an independent predictor of improved PFS (HR = 0.41, P < 0.05) (36). In addition to being a tumor burden marker, AFP reshapes the immunosuppressive microenvironment through mechanisms such as inducing immune tolerance, inhibiting dendritic cell antigen presentation, promoting Treg cell expansion, and upregulating PD-L1 expression, thereby weakening the response to immunotherapy (37). Notably, preclinical studies have shown that AFP vaccination can activate antigen-specific T cell responses and enhance anti-tumor immunity (38), providing a rationale for exploring combination strategies with immune checkpoint inhibitors in AFP-positive HCC patients. Regarding nutritional metabolism indicator, Hb <128 g/L were significantly associated with poorer outcomes (HR = 1.71, P = 0.015), consistent with findings by Jia et al. (18). This may be because anemia may activate VEGF and PDGF through hypoxia-inducible factors (HIF), thereby promoting tumor angiogenesis (39). Although the prognostic nutritional index (PNI) did not reach statistical significance in multivariate analysis, its inclusion in the model suggests a potential role of nutritional status in modulating immunotherapy response, supporting the hypothesis that nutritional interventions may improve treatment efficacy (40). Younger patients (<38 years) exhibited worse prognosis, possibly due to early hepatitis B virus infection leading to cirrhosis or more aggressive tumor biology in this population (41). Furthermore, the incorporation of systemic inflammatory markers (SII and HALP) reinforces the impact of chronic inflammation in shaping an immunosuppressive tumor microenvironment (42). While BMI and PDW did not show significant predictive value in multivariate analysis, their trends in univariate analyses warrant further validation in larger cohorts.

The current study has several limitations. (1) As a retrospective study, it is inevitably subject to selection bias. (2) The follow-up data were obtained from a single center, lacking external validation. (3) This study did not perform longitudinal comparisons of biomarkers before and after immunotherapy. Future research should incorporate serial measurements during treatment to explore the predictive value of dynamic biomarker models. (4) It is important to note that the primary objective of this study was to develop a clinically applicable prognostic model using routine parameters. While we discuss potential biological mechanisms, the complex and systematic interplay between these variables warrants further validation through prospective studies and dedicated basic science research. Therefore, there is an urgent need for randomized, multicenter, large-sample, and long-term follow-up studies to evaluate and improve the practical applicability and utility of this model.

## Conclusion

This study successfully developed and validated a prognostic model that integrates inflammation, nutrition, and tumor burden dimensions, providing a supportive tool for immunotherapy decision-making in patients with advanced HCC. However, further optimization of the model through multicenter large-sample studies is required to promote the clinical application of precision treatment strategies.

## Data Availability

The raw data supporting the conclusions of this article will be made available by the authors, without undue reservation.

## Glossary

AFP: Alpha-Fetoprotein
AGR: Albumin-to-Globulin Ratio
ALB: Albumin
ALBI: Albumin-Bilirubin Index
ALT: Alanine Aminotransferase
ANRI: Aminotransferase-to-Neutrophil Ratio Index
APRI: Aminotransferase-to-Platelet Ratio Index
AST: Aspartate Aminotransferase
AUC: The Area Under the Curve
BCLC: Barcelona Clinic Liver Cancer
BMI: Body Mass Index
CI: Confidence Interval
CT: Computed Tomography
DCA: Decision Curve Analysis
ECOG: Eastern Cooperative Oncology Group
FIB: Fibrinogen
GRIm: Modified Gustave Roussy Immune Score
GLO: Globulin
HAIC: Hepatic Arterial Infusion Chemotherapy
HALP: Hemoglobin, Albumin, Lymphocyte, and Platelet
Hb: Hemoglobin
HBsAg: Hepatitis B surface antigen
HBV: Hepatitis B Virus
HCC: Hepatocellular Carcinoma
HCV: Hepatitis C Virus
HIF: Hypoxia-Inducible Factors
HR: Hazard Ratio
HVTT: Hepatic Vein Tumor Thrombus
ICB: Immune Checkpoint Blockade
ICIs: Immune Checkpoint Inhibitors
IQR: Interquartile Range
LASSO: Least Absolute Shrinkage and Selection Operator
LDH: Lactate Dehydrogenase
LMR: Lymphocyte-to-Monocyte Ratio
MRI: Magnetic Resonance Imaging
NLR: Neutrophil-Lymphocyte Ratio
OS: Overall Survival
PDGF: Platelet-Derived Growth Factor
PDW: Platelet Distribution Width
PFS: Progression-Free Survival
PDW: Platelet Distribution Width
PLR: Platelet-to-Lymphocyte Ratio
PNI: Prognostic Nutritional Index
PVTT: Portal Vein Tumor Thrombus
ROC: Receiver Operating Characteristic
SII: Systemic Immune-Inflammation Index
TACE: Transarterial Chemoembolization
TBIL: Total Bilirubin
TKI: Tyrosine Kinase Inhibitor
TMB: Tumor Mutational Burden
VEGF: Vascular Endothelial Growth Factor
