# Supplementary material for: LASSO-empowered nomogram integrating nutritional-inflammatory-tumor characteristics predicts immunotherapy outcomes in advanced HCC: Large retrospective cohort
Source: Front Immunol. 2025 Dec 1;16:1626940. doi: 10.3389/fimmu.2025.1626940 (PMC12702932; doi:10.3389/fimmu.2025.1626940)
Supplement: Supplementary file 1 [file DataSheet1.docx]

**Supplementary Figure 1. Kaplan-Meier Survival Curves for PFS and OS in enrolled HCC patients, with the respective median values highlighted.**


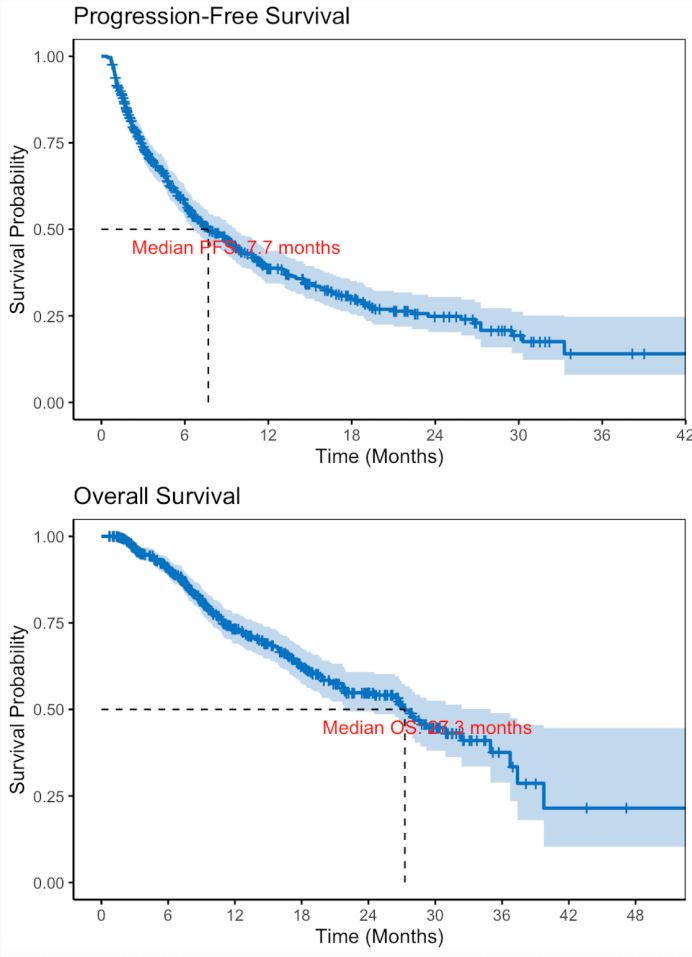


Abbreviations: HCC, Hepatocellular Carcinoma; OS, Overall Survival; PFS, Progression-Free Survival.

**Supplementary Figure 2. Density distribution of various biomarkers with optimal cutoff for survival stratification.**


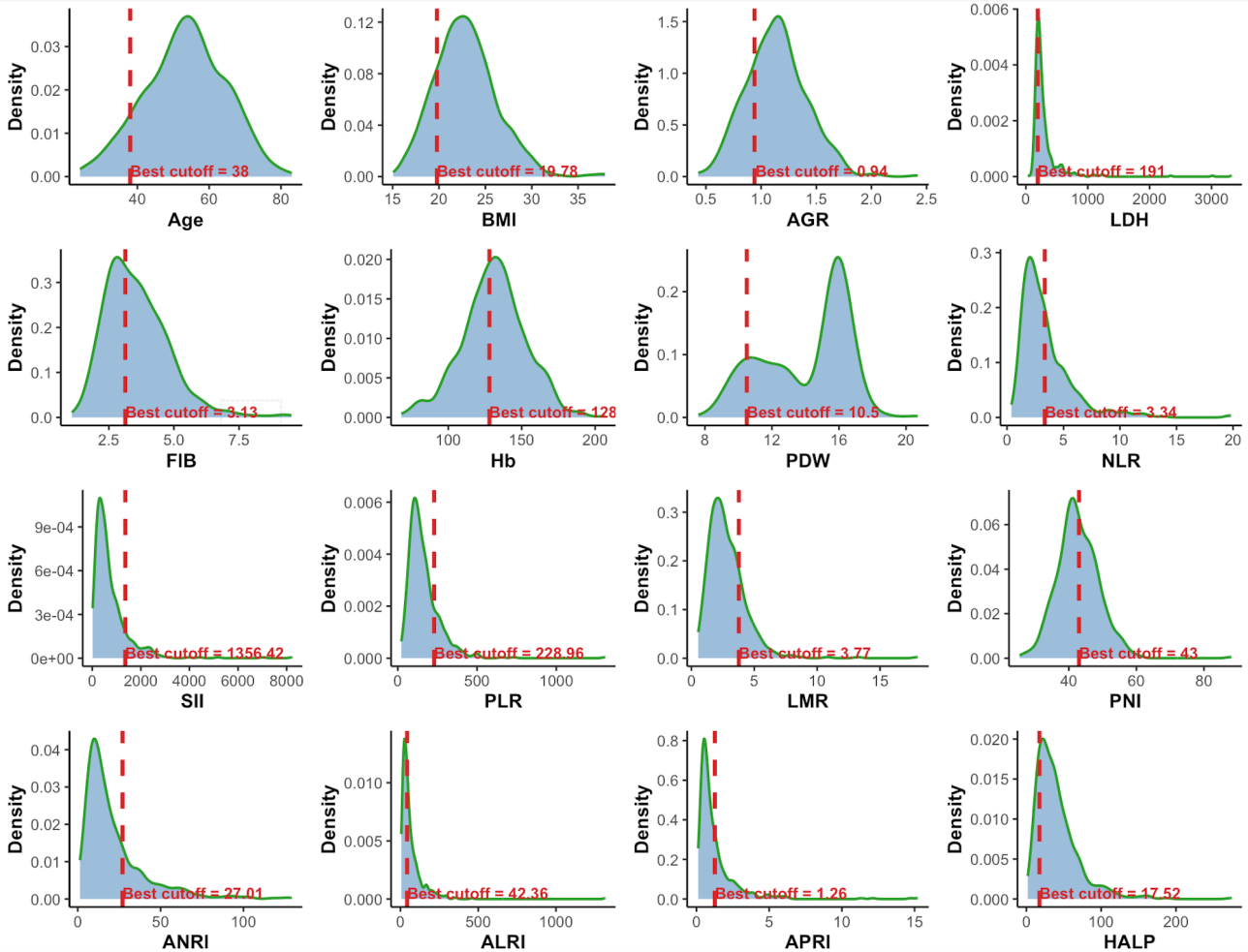


Abbreviations: AFP, Alpha-fetoprotein; AGR, Albumin-to-Globulin Ratio; ALRI, Aminotransferase-to -lymphocyte Ratio Index; ANRI, Aminotransferase-to-neutrophil Ratio Index; APRI, Aminotransferase-to-platelet Ratio Index ; BMI, Body Mass Index; FIB, Fibrinogen; HALP, Hemoglobin, Albumin, Lymphocyte, and Platelets; Hb, Hemoglobin; LDH, Lactate Dehydrogenase; LMR, Lymphocyte-to-Monocyte Ratio; NLR, Neutrophil-Lymphocyte Ratio; PDW, Platelet Distribution Width; PLR, Platelet-to-Lymphocyte Ratio; SII, Systemic Immune-Inflammation Index; PNI, Prognostic Nutritional Index.

**Supplementary Figure 3. Overall Survival stratified by various biomarkers based on the optimal cutoff.**


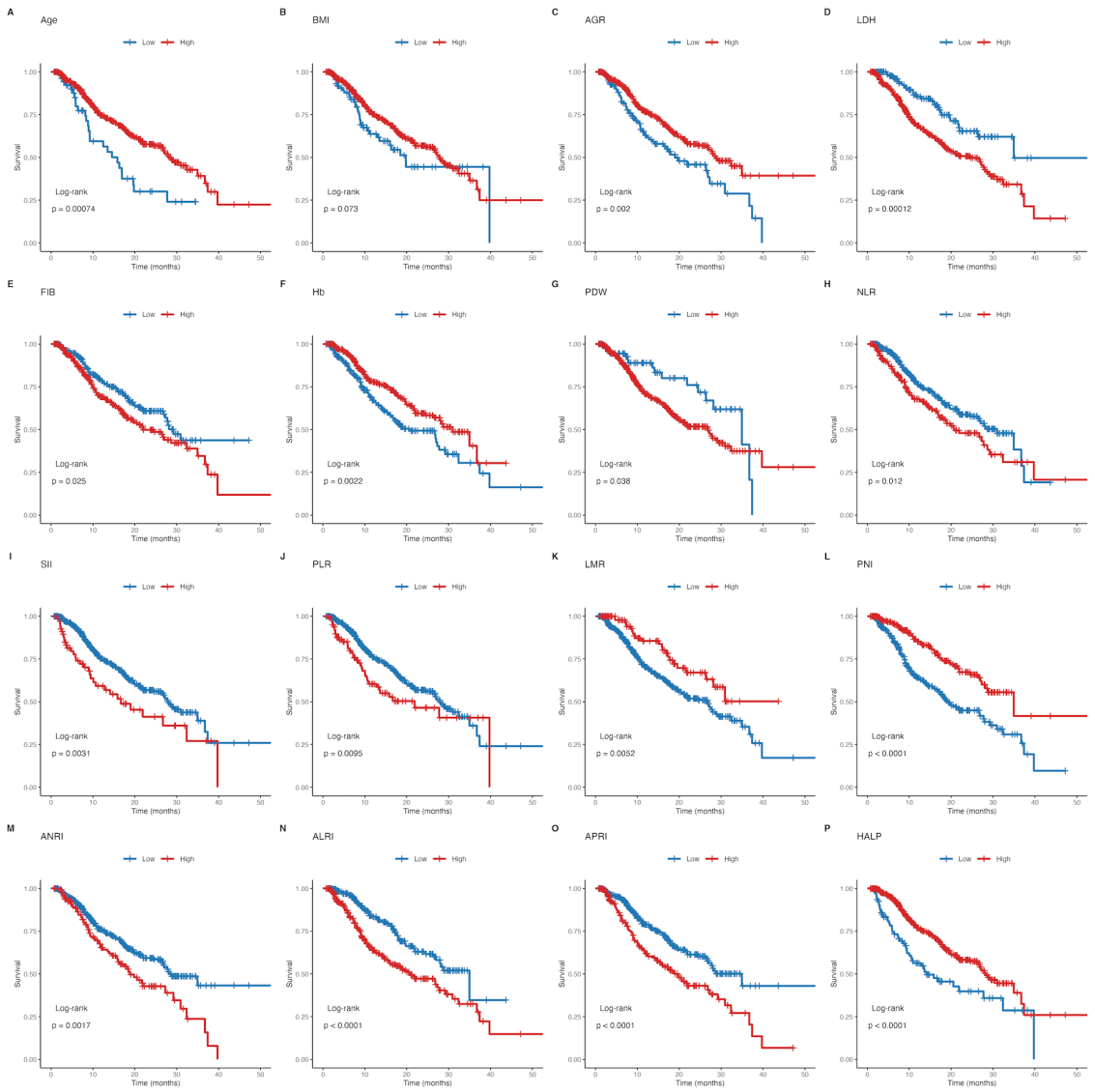


Abbreviations: AFP, Alpha-fetoprotein; AGR, Albumin-to-Globulin Ratio; ALRI, Aminotransferase-to -lymphocyte Ratio Index; ANRI, Aminotransferase-to-neutrophil Ratio Index; APRI, Aminotransferase-to-platelet Ratio Index; BMI, Body Mass Index; FIB, Fibrinogen; HALP, Hemoglobin, Albumin, Lymphocyte, and Platelets; Hb, Hemoglobin; LDH, Lactate Dehydrogenase; LMR, Lymphocyte-to-Monocyte Ratio; NLR, Neutrophil-Lymphocyte Ratio; PDW, Platelet Distribution Width; PLR, Platelet-to-Lymphocyte Ratio; SII, Systemic Immune-Inflammation Index; PNI, Prognostic Nutritional Index.

**Supplementary Figure 4. Survival Analysis of OS based on risk stratification.**


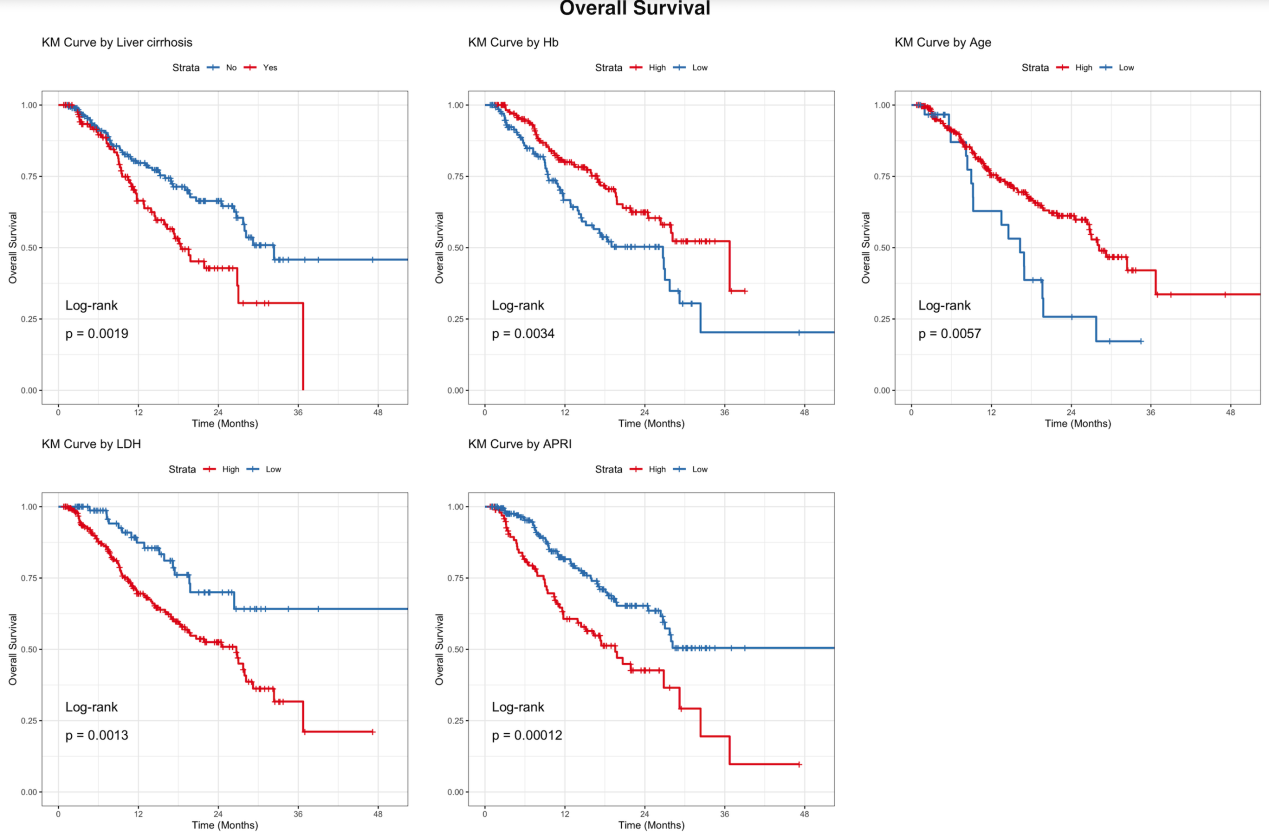


Abbreviations: APRI, Aminotransferase-to-platelet Ratio Index; Hb, Hemoglobin; LDH, Lactate Dehydrogenase; OS, Overall Survival.

**Supplementary Figure 5. Histogram of coefficients for selected features.**


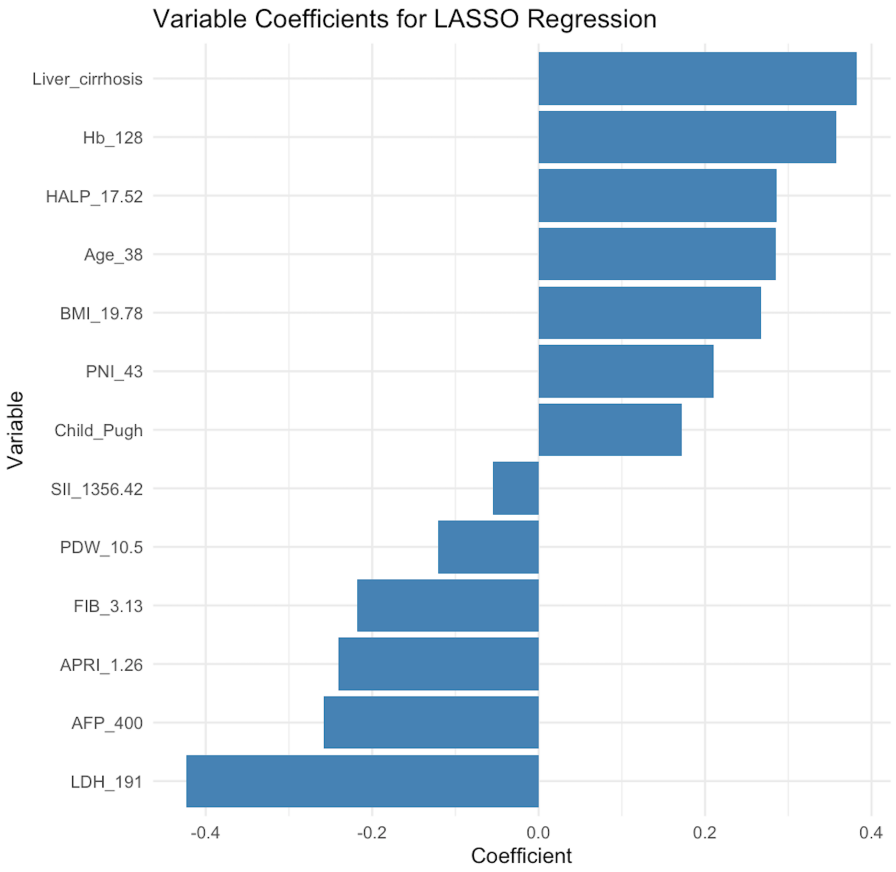


Abbreviations: AFP, alpha-fetoprotein; APRI, Aminotransferase-to-platelet Ratio Index; BMI, Body Mass Index; FIB, Fibrinogen; HALP, Hemoglobin, Albumin, Lymphocyte, and Platelets; Hb, Hemoglobin; LASSO, Least Absolute Shrinkage and Selection Operator; LDH, Lactate Dehydrogenase; PDW, Platelet Distribution Width; PNI, Prognostic Nutritional Index; SII, Systemic Immune-Inflammation Index.

**Supplementary Figure 6. ROC Curves for predicting 3-, 6-, 12-, and 24-Month Overall Survival probabilities in different treatment groups.**


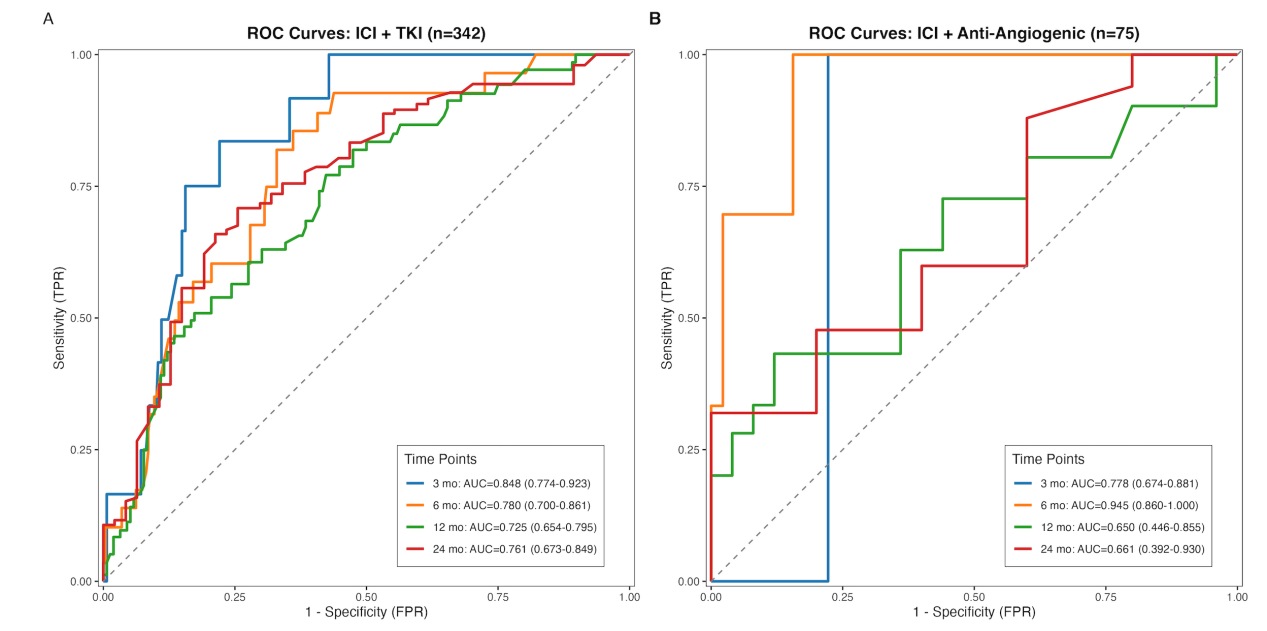


Abbreviations: AUC, Area Under the Curve；ICI, Immune Checkpoint Inhibitor; ROC, Receiver Operating Characteristic; TKI, Tyrosine Kinase Inhibitor.

**Supplementary Figure 7. Survival analysis across different treatment regimens.**


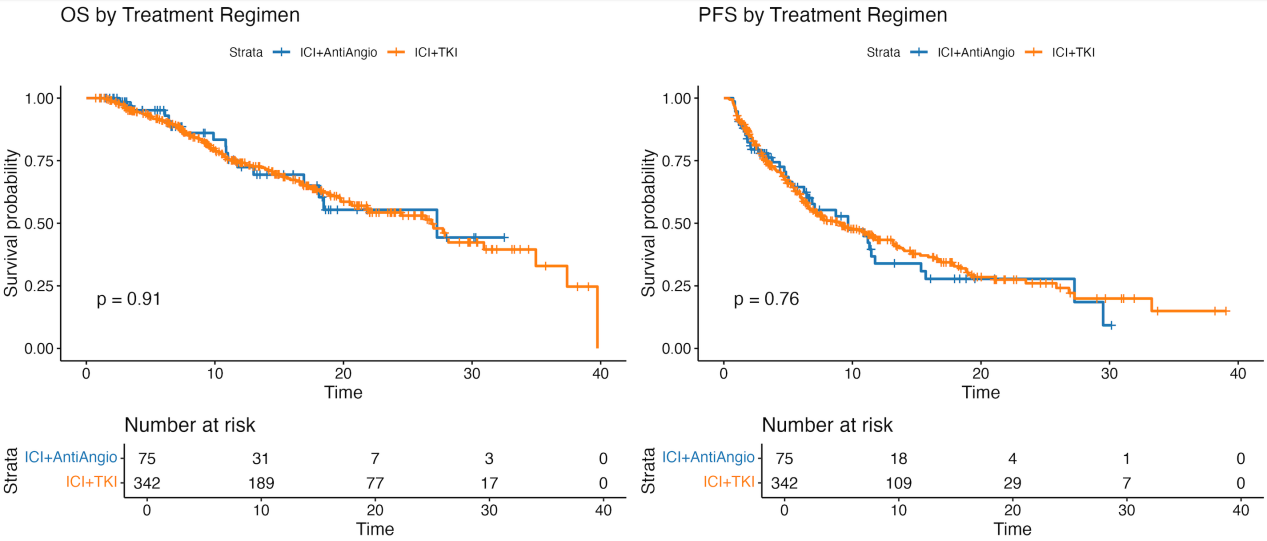


Abbreviations: ICI, Immune Checkpoint Inhibitor; OS, Overall Survival; PFS, Progression-Free Survival; TKI, Tyrosine Kinase Inhibitor.

**Supplementary Figure 8. Validation of the prognostic model in HBV-Positive patients: (A) time-dependent ROC curves, (B) calibration plot, and (C) decision curve analysis.**


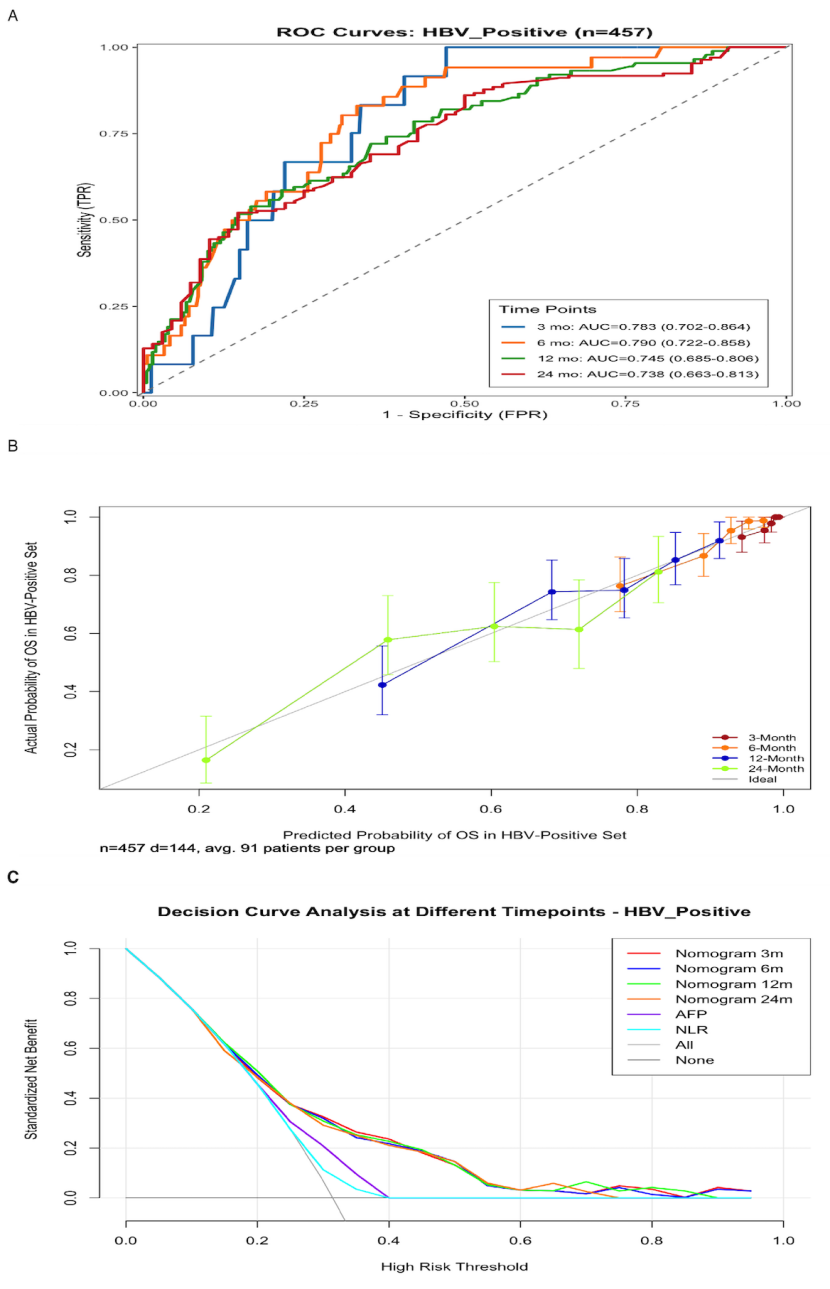


Abbreviations: AUC, Area Under the Curve; HBV, Hepatitis B Virus; OS, Overall Survival; ROC, Receiver Operating Characteristic.

**Supplementary Table 1.Coefficients of variables selected by LASSO regression.**

| Variable | Coefficient |
| --- | --- |
| Age≤38 | 0.285398183825753 |
| BMI≤19.78 | 0.267301268039745 |
| Liver_cirrhosis | 0.382184669022061 |
| Child Pugh B | 0.172396267306724 |
| AFP≤400 | -0.258023427 |
| LDH≤191 | -0.423095927 |
| FIB≤3.13 | -0.217591166 |
| Hb≤128 | 0.357307249553105 |
| PDW≤10.5 | -0.120660711 |
| SII≤1356.42 | -0.054882588 |
| PNI≤43 | 0.210740694413824 |
| APRI≤1.26 | -0.241054379 |
| HALP≤17.52 | 0.286338463718908 |

Abbreviations: AFP, alpha-fetoprotein; APRI, Aminotransferase-to-platelet Ratio Index; BMI, Body Mass Index; FIB, Fibrinogen; HALP, Hemoglobin, Albumin, Lymphocyte, and Platelets; Hb, Hemoglobin; LASSO, Least Absolute Shrinkage and Selection Operator; LDH, Lactate Dehydrogenase; PDW, Platelet Distribution Width; PNI, Prognostic Nutritional Index; SII, Systemic Immune-Inflammation Index.
